# GDF15 Is Elevated in Conditions of Glucocorticoid Deficiency and Is Modulated by Glucocorticoid Replacement

**DOI:** 10.1210/clinem/dgz277

**Published:** 2019-12-19

**Authors:** Audrey Melvin, Dimitrios Chantzichristos, Catriona J Kyle, Scott D Mackenzie, Brian R Walker, Gudmundur Johannsson, Roland H Stimson, Stephen O’Rahilly

**Affiliations:** 1 MRC Metabolic Diseases Unit, Wellcome Trust-MRC Institute of Metabolic Science, University of Cambridge, Addenbrookes Treatment Centre, UK; 2 Department of Internal Medicine and Clinical Nutrition, Institute of Medicine at Sahlgrenska Academy, University of Gothenburg, Gothenburg, Sweden; 3 Department of Endocrinology-Diabetes-Metabolism, Sahlgrenska University Hospital, Gothenburg, Sweden; 4 University/ BHF Centre for Cardiovascular Science, Queen’s Medical Research Institute, University of Edinburgh, UK; 5 Institute of Genetic Medicine, Newcastle University, Newcastle upon Tyne, UK

**Keywords:** glucocorticoids, adrenal insufficiency, GDF15

## Abstract

**Context:**

GDF15 is a stress-induced hormone acting in the hindbrain that activates neural circuitry involved in establishing aversive responses and reducing food intake and body weight in animal models. Anorexia, weight loss, nausea and vomiting are common manifestations of glucocorticoid deficiency, and we hypothesized that glucocorticoid deficiency may be associated with elevated levels of GDF15.

**Objective:**

To determine the impact of primary adrenal insufficiency (PAI) and glucocorticoid replacement on circulating GDF15 levels.

**Methods and Results:**

We measured circulating concentrations of GDF15 in a cohort of healthy volunteers and Addison’s disease patients following steroid withdrawal. Significantly higher GDF15 (mean ± standard deviation [SD]) was observed in the Addison’s cohort, 739.1 ± 225.8 pg/mL compared to healthy controls, 497.9 ± 167.7 pg/mL (*P* = 0.01). The effect of hydrocortisone replacement on GDF15 was assessed in 3 independent PAI cohorts with classical congenital adrenal hyperplasia or Addison’s disease; intravenous hydrocortisone replacement reduced GDF15 in all groups. We examined the response of GDF15 to increasing doses of glucocorticoid replacement in healthy volunteers with pharmacologically mediated cortisol deficiency. A dose-dependent difference in GDF15 (mean ± SD) was observed between the groups with values of 491.0 ± 157.7 pg/mL, 427.0 ± 152.1 pg/mL and 360 ± 143.1 pg/mL, in the low, medium and high glucocorticoid replacement groups, respectively, *P* < .0001.

**Conclusions:**

GDF15 is increased in states of glucocorticoid deficiency and restored by glucocorticoid replacement. Given the site of action of GDF15 in the hindbrain and its effects on appetite, further study is required to determine the effect of GDF15 in mediating the anorexia and nausea that is a common feature of glucocorticoid deficiency.

GDF15 (then called MIC-1) was first identified in 1997 as a novel transcript from a macrophage cell line and classified as a member of the transforming growth factor beta (TGF-β) superfamily (1). However, in humans, GDF15 is widely expressed with highest levels seen in placental trophoblasts followed by kidney, bladder, prostate, gastrointestinal, pancreatic, lung, liver, and adipose tissue (2). GDF15 has been proposed as a marker of cellular stress with elevated circulating levels observed in a number of physiological and pathological states (3). Conditions where increased circulating GDF15 have been reported include but are not limited to pregnancy, exercise, aging, renal failure, cardiac failure, chronic inflammatory disease, neoplasia, and mitochondrial diseases as well as cytotoxic chemotherapy and ionizing radiation (3). Aligned with the view that GDF15 represents a marker of cellular stress, increased expression and secretion of GDF15 has been demonstrated when the cellular integrated stress response (ISR) pathway is activated (4, 5, 6) suggesting that GDF15 represents an endocrine arm of the cellular ISR. Key advances in our understanding of the functional biology of GDF15 have come from the observations that (i) overexpression of GDF15 in mice resulted in weight loss and reduced energy intake (7); (ii) GDF15 knockout mice have increased body mass and energy intake compared to wild-type counterparts (8); and (iii) GDF15 seemingly mediates these effects on energy intake and body mass through the hind brain (9). Subsequently, glial-derived neurotrophic factor receptor alpha-like (GFRAL) was identified as the ligand binding component of the heterodimeric receptor for GDF15 (10–13). GFRAL expression is highly specific to the area postrema and nucleus tractus solitarius of the hindbrain where it is required to mediate the weight lowering effects of GDF15. There is compelling evidence in rodents and non-human primates that manipulation of the GDF15-GFRAL pathway induces an anorectic effect (10–13). At present, evidence supporting the anorectic potential of GDF15 in humans is largely limited to observational studies. Increased GDF15 in a range of cancer types has been reported to associate with weight loss and is implicated in the pathogenesis of cancer cachexia (7, 14–16). Similar associations have been observed in cardiac failure where higher GDF15 serves not only as a biomarker for mortality risk but there is an inverse relationship seen between GDF15 and body mass index (BMI) (17). In chronic kidney disease (CKD), GDF15 is also predictive of mortality. Higher levels of GDF15 have been reported in dialysis patients who had protein energy wasting, while among CKD patients increasing tertiles of GDF15 are associated with decreasing BMI (18, 19). Therapeutically targeting the GDF15–GFRAL axis has gained considerable attention in the management of anorexia and cachexia associated with cancer and other chronic diseases (20).

However, it is not clear why a stress induced hormone such as GDF15 should primarily reduce energy intake. It has been proposed that GDF15 may have evolved originally to provide a signal to the organism that a harmful toxin has been ingested, with the brainstem-derived signal serving in the acute setting to reduce continued ingestion and in the longer term to avoidance upon future encounters (21). In support of this hypothesis is genetic (22) and biochemical (23) evidence in humans implicating GDF15 in the pathogenesis of the nausea and vomiting of pregnancy, including hyperemesis gravidarum. Further, at least at pharmacological doses, recombinant GDF15 induces a conditioned taste aversion in rodents (4). Moreover, the emetic potential of GDF15 was recently illustrated in response to administration of the peptide to musk shrews (24). Collectively, these observations suggest that GDF15 has aversive properties and this may underpin its effect on energy intake.

Primary adrenal insufficiency (PAI) is a rare condition with a reported prevalence of between 100 to 140 cases per million and represents a potentially life-threatening illness that is characterized by the impaired ability of the adrenal cortex to produce sufficient amounts of glucocorticoid and/or mineralocorticoid (25). Glucocorticoids are produced and secreted under the control of adrenocorticotropin and regulate a number of basal and stress-mediated physiological responses that include inflammatory, metabolic, and hemodynamic processes (26, 27) while mineralocorticoids are under the regulation of the renin–angiotensin system and play a key role in fluid and electrolyte balance (28). Although a number of different etiologies are responsible, patients with PAI share common clinical features attributable to deficiency of the respective steroid hormones (25, 29). To date, there are no reports on the effect of glucocorticoid deficiency on circulating GDF15. Among individuals diagnosed with PAI symptoms of nausea, vomiting, and anorexia are reported in greater than two-thirds of those affected and responsive to hormone replacement (29). In some instances, the severity of such symptoms has led to the misdiagnosis of PAI as anorexia nervosa (30) or presented as intractable vomiting (31). We hypothesized that GDF15 would be increased in the setting of glucocorticoid deficiency and may contribute to the nausea/vomiting/anorexia phenotype frequently observed in adrenal insufficiency. To explore this, we measured circulating GDF15 in volunteers with PAI and pharmacologically induced glucocorticoid deficiency and determined its response to glucocorticoid replacement.

## Methodology

### Cohort 1: Addison’s disease

The study was approved by the Ethics Review Board of the University of Gothenburg, Sweden (permit no. 374-13, August 8, 2013) and written informed consent was obtained from all patients before participation. This was a randomized cross-over, single-blinded trial including subjects with Addison’s disease who received in random order intravenous hydrocortisone infusion in isotonic saline in a circadian pattern or the same volume of isotonic saline for 22 hours, at least 2 weeks apart. Dosing of the hydrocortisone infusion was adapted from Kerrigan et al (32). The intravenous hydrocortisone (Solu-Cortef) infusion was initiated from 0900h at starting at a dose of 0.024 mg/kg/h before being decreased gradually to achieve a dose of 0.008 mg/kg/h by 0000h, at which point the dose was increased to 0.03mg/kg/h and maintained until blood sampling at 0700h. Daily replacement doses of hydrocortisone were withdrawn for 17 hours prior to initiation of the infusion. Subjects omitted their usual fludrocortisone dose (when prescribed) the day before and during each intervention. Ten subjects (4 women and 6 men) with Addison’s disease with a mean ± SD age of 46.6 ± 9.4 years, BMI of 25.6 ± 2.5 kg/m^2^ and a median disease duration of 23.5 years (min–max, 1–33) were included in the study. The median daily replacement dose of hydrocortisone prior to the study was 30 mg (min–max, 20–30).

### Cohort 2: Addison’s disease

Full details of this protocol have been published previously (33). Ethical approval from the South East Scotland Research Ethics committee and informed consent were obtained from all participants. In brief, subjects with Addison’s disease attended the Edinburgh Clinical Research Facility after an overnight fast and having omitted their usual glucocorticoid morning dose. Subjects omitted their usual fludrocortisone dose (when prescribed) the day before and on the morning of their visits. Serum samples obtained from eight subjects (6 female, 2 male, mean ± SD age 51.6 ± 14.8 years and BMI 25.1 ± 3.6 kg/m^2^) at baseline *t *= 0 min and following 320 min of a 3-step intravenous infusion of 9,11,12,12-[^2^H]_4_-hydrocortisone were analyzed for GDF15. Mean ± SD cortisol concentrations at baseline were 28.2 ± 41.0 nmol/L and by *t* + 320 min deuterated cortisol concentrations were 410.9 ± 255.7 nmol/L.

### Cohort 3: Congenital adrenal hyperplasia

Nine subjects (7 female, 2 male, mean ± SD age 38.7 ± 12.2 years and BMI 32.1 ± 12.3 kg/m^2^) with classic congenital adrenal hyperplasia (CAH) secondary to 21-hydroxylase deficiency were recruited to a randomized double-blind crossover study comparing placebo and hydrocortisone infusion as part of a separate study protocol. Ethical approval from the South East Scotland Research Ethics committee and informed consent were obtained from all participants. Subjects attended the Edinburgh Clinical Research Facility after overnight fast and omitting their usual glucocorticoid treatment from 1800h onwards the previous day, although subjects still took their usual fludrocortisone dose that morning if prescribed. Subjects received either an intravenous infusion of hydrocortisone for 300 min (following a 2.6 μmol bolus hydrocortisone was infused at 51.5 nmol/min from *t* = 0 min until *t* + 150 min, then following a further 2.6 μmol intravenous bolus the hydrocortisone was infused at 103 nmol/min until the end of the protocol) or placebo (0.9% sodium chloride). Plasma samples obtained at baseline and at the end of the infusion during both visits were analyzed for GDF15 concentrations. Mean ± SD circulating cortisol concentrations were 32.5 ± 30.4 nmol/L at baseline and rose to 493.9 ± 79.8 nmol/L at *t *+ 300 min of the hydrocortisone infusion, while cortisol levels were 26.8 ± 23.4 nmol/L at baseline and 20.4 ± 18.1 nmol/L at *t* + 300 min of the placebo infusion.

### Cohort 4: Pharmacological cortisol deficiency

Full details of this protocol have been published previously (34). Ethical approval from the South East Scotland Research Ethics committee and informed consent was obtained from all participants. In brief, 20 male subjects (Mean ± SD age 33.4 ± 15.6 years and BMI 23.8 ± 1.6 kg/m^2^) were recruited to a randomized double-blind crossover study comparing the effects of low, medium, and high cortisol concentrations. Subjects attended the Edinburgh Clinical Research Facility on 3 separate occasions after overnight fast. Subjects all received the 11-beta-hydroxylase inhibitor metyrapone, 1 g orally at 2200h the night before and at 0700h and 1100h on the morning of each assessment to inhibit endogenous cortisol synthesis. Subjects received either placebo (low cortisol phase) or hydrocortisone tablets (10 mg at 2200h the night before and 5 mg at 0700h on medium cortisol phase, 20 mg at 2200h the night before and 10 mg at 0700h on high cortisol phase) in random order. Upon arrival at the research facility, subjects received an intravenous infusion of either 0.9% sodium chloride (placebo phase) or hydrocortisone (at 0.025 mg/kg/h following a 0.04 mg/kg bolus [medium cortisol phase] or at 0.12 mg/kg/h following a 0.18 mg/kg bolus [high cortisol phase]) for 345 min. Plasma samples obtained at 285 min of the infusion were analyzed for GDF15. Mean ± SD cortisol concentrations at 285 min of the infusion were 170.2 ± 54.0 (low), 464.9 ± 90.2 (medium), and 1479.4 ± 246.6 (high) nmol/L for the respective phases.

### Healthy volunteer study

The study was approved by the Cambridge central research ethics committee (REC 06/Q0108/84) and written informed consent were obtained from all participants prior to participation in the study. Ten participants from the healthy volunteer study were selected as an age, sex, and BMI matched control group for participants from cohort 1 with Addison’s disease. Exclusion criteria were (i) age <17 or >65 years; (ii) pregnancy or breast feeding; (iii) any current or past medical disorder that could affect the reference measurements; (iv) any current or previous medical disorder that could influence a participant’s ability to follow the study protocols safely and effectively; (v) any current or previous medication that affect the reference measurements; (vi) current or previous drug or alcohol abuse; (vii) current smoking habit; and (viii) any concern that a participant may not understand study protocols sufficiently to give informed consent. Volunteers attended the National Institute for Health Research (NIHR) Cambridge Clinical Research Facility for assessment where study bloods were taken from the participants at 0800h following an overnight fast. GDF15 and cortisol were measured on serum collected from the 10 participants.

### Biochemical assays

#### Cortisol.

*Cohort 1, 2, 3*: Endogenous and deuterated cortisol concentrations were analyzed by liquid-chromatography tandem mass spectrometry (ABSciex Qtrap 5500 with Waters Acquity UPLC; Column: ACE Excel C18-AR 2.1×150mm) as previously described (33) at the Mass Spectrometry Core Laboratory, Centre for Cardiovascular Science, Queen’s Medical Research Institute in Edinburgh, UK. *Cohort 4*: Cortisol was measured by radioimmunoassay as previously described (34). *Healthy volunteer study.* Serum cortisol was measured using LC-MS/MS at Cambridge university hospital NHS foundation trust. Serum proteins were precipitated and cortisol released from its binding proteins using ZnSO_4_ precipitation. The sample was processed using online solid phase extraction then HPLC separation using a pheny-hexyl column. Deuterated cortisol was used as an internal standard. Mass transitions used are 363.3/121.2 (quantifier) 363.3/327.3 (qualifier) and 367.3 /121.1 (internal standard). An APISciex 550 mass spectrometer was used with APCI ionization in positive ion mode.

#### GDF15.

GDF15 measurements on all participants’ serum or plasma was undertaken at the Cambridge Biochemical Assay Laboratory, University of Cambridge, using antibodies and standards from R&D Systems (R&D Systems Europe, Abingdon UK). GDF15 was measured using a microtiter plate-based 2-site electrochemiluminescence immunoassay with the MesoScale Discovery assay platform (MSD, Rockville, Maryland, US).

### Statistical analysis

Data are presented as mean ± SD unless otherwise stated. Data were analyzed using Graphpad Prism® Version 6. Differences between the means were compared using paired and unpaired 2 sample student *t*-tests as indicated. A one-way analysis of variance and post-hoc Tukey test was used to test differences between means where >2 groups were present. The null hypothesis was rejected at *P* < .05.

## Results

### Primary adrenal insufficiency is associated with increased GDF15 levels

**Figure 1.**
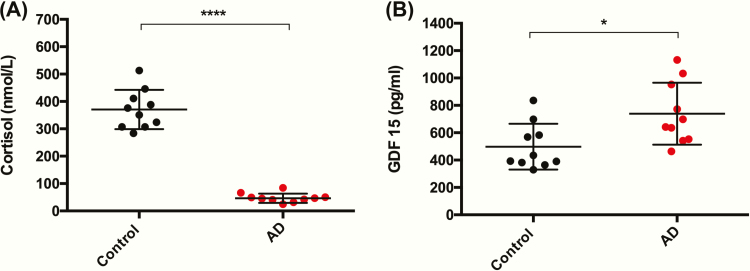
Circulating GDF15 is increased in primary adrenal insufficiency. **(A)** Early morning serum cortisol levels are significantly lower in 10 patients with Addison’s disease (AD) following withdrawal of maintenance glucocorticoid therapy when compared to a cohort of healthy matched control participants (*n* = 10). **(B)** Serum GDF15 measured in parallel with cortisol sampling was significantly higher within the steroid deficient Addison’s disease cohort than in the control group. Data presented as mean ± standard deviation and compared using an unpaired *t*-test. *P < .05 and ****P < .0001.

To determine whether circulating levels of GDF15 among patients with PAI differed significantly from those of healthy volunteers, we compared GDF15 and cortisol measurements from patients with PAI due to Addison’s disease in cohort 1 (male *n* = 6, female *n* = 4, age 46.4 ± 9.4 years, BMI 25.6 ± 2.5 kg/m^2^) to a matched control group from the healthy volunteer study (male *n* = 6, female *n* = 4, age 43.6 ± 11.7 years, BMI 25.4 ± 3.6 kg/m^2^). Patients with Addison’s disease had their glucocorticoid and mineralocorticoid therapy withdrawn for at least 39 hours (see methods—placebo group cohort 1) prior to measurement of morning cortisol and GDF15. As expected, serum cortisol levels were significantly lower among patients with Addison’s disease than the matched control group, 46.5 ± 16.9 nmol/L versus 370.7 ± 71.55 nmol/L (*P* < .0001), respectively (Fig. 1A). We observed significantly higher levels of GDF15 in the Addison’s disease cohort who were glucocorticoid deficient than in the matched healthy volunteer cohort 739.1 ± 225.8 pg/mL versus 497.9 ± 167.7 pg/mL (*P* = .01), respectively (Fig. 1B).

### Glucocorticoid replacement reduces circulating GDF15 in primary adrenal insufficiency

**Figure 2.**
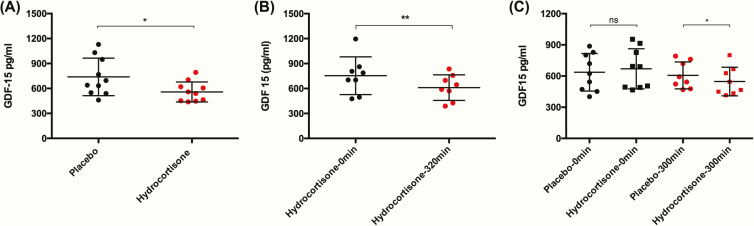
Hydrocortisone replacement reduces GDF15 in primary adrenal insufficiency. **(A)** Ten adult volunteers with Addison’s disease who had glucocorticoid therapy withdrawn demonstrated significantly lower GDF15 levels following a 22-h intravenous infusion of hydrocortisone (red circles) compared to the equivalent infusion of normal saline (black circles). **(B)** GDF15 levels reduced significantly from baseline measurements following infusion of hydrocortisone in 8 participants with Addison’s disease following withdrawal of glucocorticoid therapy. **(C)** Among 9 participants with CAH whose glucocorticoid therapy was withdrawn, baseline GDF15 levels did not differ significantly between placebo (black circles) and hydrocortisone (black squares) visits. GDF15 levels were significantly lower in participants (*n* = 8) who received the hydrocortisone infusion (red squares) when compared to those receiving placebo (red circles). Data expressed as mean ± standard deviation and compared using a paired *t*-test, *P < .05 and **P < .01.

To determine the effect of correcting glucocorticoid deficiency on GDF15, ten participants (male = 6, female = 4) with Addison’s disease (cohort 1) were assessed on 2 occasions. Glucocorticoid treatment was withdrawn for 17 hours before they were randomized to receive a hydrocortisone infusion mimicking physiological glucocorticoid action from 0900h to 0700h or an infusion of normal saline in a crossover design. Circulating cortisol levels were significantly higher at 0700h following the glucocorticoid intervention compared to placebo 362 ± 292.9 nmol/L versus 46.5 ± 16.9 nmol/L (*P* = .006), respectively. GDF15 was significantly increased during the placebo infusion at 739.1 ± 225.8 pg/mL when compared to the hydrocortisone infusion 558.5 ± 119.9, *P* = .017 (Fig. 2A). To evaluate the reproducibility of the observation that glucocorticoid replacement in PAI was associated with a reduction in circulating GDF15 we assessed 2 independent PAI cohorts comprising of patients with Addison’s disease (cohort 2) or classical CAH (cohort 3). Maintenance glucocorticoid therapy was withdrawn for at least 18 hours in the Addison’s cohort, GDF15 was measured at baseline and following 320 min of hydrocortisone infusion. There was a significant reduction in circulating GDF15 following the infusion (610.4 ± 153.4 pg/mL) when compared to baseline (753.8 ± 226.4 pg/mL, *P* = .008) (Fig. 2B). The CAH cohort was studied on 2 occasions following the withdrawal of maintenance glucocorticoid replacement therapy (for at least 14 h prior to attendance), each participant received an isotonic saline or hydrocortisone infusion over 300 min. Baseline GDF15 levels did not differ significantly between the placebo (659.1 ± 177.7 pg/mL) and glucocorticoid (667.0 ± 206.0 pg/mL) study visits (*P* = .760). Circulating GDF15 levels were significantly lower when participants (*n* = 8) received a 300-min infusion of hydrocortisone (547.0 ± 137.0 pg/mL) than the normal saline placebo infusion (606.0 ± 129.8 pg/mL), *P* = .014 (Fig. 2C).

### Glucocorticoid replacement demonstrates a dose-dependent relationship with GDF15

**Figure 3.**
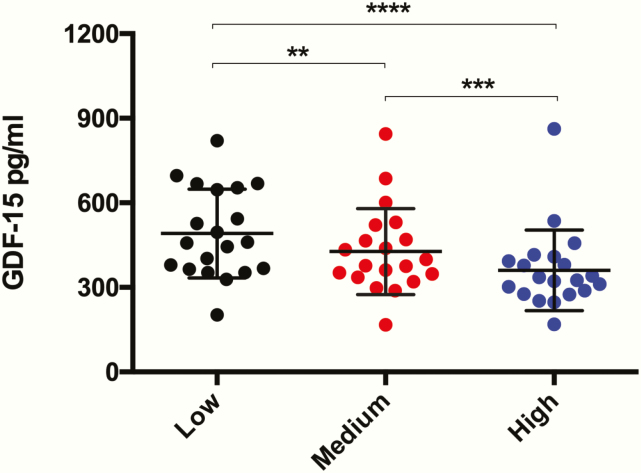
Dose effect of hydrocortisone replacement on circulating GDF15. Serum GDF15 was measured in *n* = 20 healthy male volunteers with metyrapone inhibited endogenous cortisol synthesis who received low, medium, and high dose hydrocortisone replacement on three separate occasions. Exposure to increasing doses of hydrocortisone was associated with significantly lower GDF15 levels. Data expressed as mean ± standard deviation and compared using a one-way analysis of variance and post-hoc Tukey test, **P < .01, ***P < .001, ****P < .0001.

We hypothesized that a dose-dependent relationship existed between glucocorticoid replacement in PAI and GDF15 levels. To explore this, we determined the effect of low, medium, or high dose hydrocortisone replacement in 20 healthy male volunteers whose endogenous cortisol production had been pharmacologically inhibited by metyrapone (cohort 4). We observed a significant and progressive reduction in GDF15 in response to increasing hydrocortisone doses (low dose = 491.1 ± 157.6 pg/mL; medium dose = 427.2 ± 152.2 pg/mL; high dose = 360.3 ± 143.1 pg/mL; analysis of variance *P* < .0001) (Fig. 3).

## Discussion

Our hypothesis that glucocorticoid may modulate GDF15 levels was based on two previous lines of evidence. First, glucocorticoids have been reported to reduce GDF15 gene expression (35, 36). Second, glucocorticoids have well established antiemetic properties, which are used clinically, particularly in the context of cytotoxic chemotherapy and radiotherapy, both of which are known to cause elevations in circulating GDF15 (37, 38). Despite a paucity of research relating to the mechanisms of nausea and vomiting in adrenal insufficiency, it is well established that these symptoms are responsive to glucocorticoid replacement (29). In this study, we demonstrate that circulating GDF15 levels are increased in patients with PAI temporarily deprived of glucocorticoid replacement therapy. In 3 independent cohorts, we demonstrated that short-term physiological glucocorticoid replacement in patients with different forms of PAI led to a reduction in circulating GDF15. Importantly, the relationship between glucocorticoid replacement and GDF15 appears to be dose dependent. Interestingly, GDF15 has been reported to have a diurnal rhythm with a peak at ~0000h and nadir at ~1200h (39). Might this be entrained by the normal circadian rhythm of cortisol secretion? The findings in our study indicate that GDF15 levels were significantly lower when cortisol levels were artificially maintained at ~400 nmol/L (similar to physiological “peak” levels) vs ~150 nmol/L (similar to physiological “nadir” levels) is at least compatible with this hypothesis. The time lag between peak cortisol and the nadir of GDF15 would be expected if cortisol is having an effect on GDF15 gene expression. Further insight may be gained from the measurement of circulating GDF15 levels in human populations with disrupted circadian rhythms.

Although we did not directly address the issue in these studies it is interesting to speculate whether GDF15 might play a role in the well-established effects of glucocorticoids on appetite and energy intake outside the setting of adrenal insufficiency, including, for example, in states of endogenous or pharmacological glucocorticoid excess (40). These effects of glucocorticoids accumulate with time. However, a limitation of our studies is that we only studied acute manipulations of glucocorticoids and for ethical reasons we could only study patients with short-term withdrawal of therapy. An additional limitation of our studies is that we did not formally address the contribution of mineralocorticoid deficiency to GDF15 levels and the CAH cohort continued to take prescribed mineralocorticoid replacement during the study. However, it is worth noting that anorexia, weight loss, nausea, and vomiting are characteristic features of untreated isolated adrenocorticotropin deficiency, a condition that selectively lowers glucocorticoid (41) rather than mineralocorticoid levels.

Despite pharmacological replacement of the glucocorticoid and mineralocorticoid deficiencies, concerns remain regarding quality of life, cardiometabolic and bone health among patients affected by PAI (42). In clinical practice there is considerable heterogeneity in glucocorticoid and mineralocorticoid replacement regimens used to treat PAI (43, 44). These observations fuel interest in determining the optimal glucocorticoid replacement strategy of patients with PAI (45) as the current empiric approaches particularly to glucocorticoid dosing cannot account for the many factors which influence hormone action at its target tissue. Our study did not extend to exploring the tissue/s from which increased circulating GDF15 was produced. In principle GDF15 may be considered as target tissue derived circulating biomarker of glucocorticoid exposure. In practice, however, the utility of GDF15 as a biomarker of tissue exposure to glucocorticoid is likely hampered by the fact GDF15 is also influenced by a wide range of physiological and pathological states. This was exemplified in one participant in the pharmacologically induced glucocorticoid deficiency cohort (Participant no. 6 (46)) who did not modulate their GDF15 level in response to increasing doses of hydrocortisone unlike the other participants.

In conclusion, we have demonstrated that circulating GDF15 levels are elevated in untreated PAI patients compared to matched healthy controls and that glucocorticoid replacement corrects the elevated GDF15 levels. GDF15 responds to glucocorticoid replacement in a dose-dependent manner. Further investigation of the causal nature of the relationship between glucocorticoid deficiency, elevated circulating levels of GDF15 and symptoms of anorexia, weight loss, nausea and vomiting is warranted. Such studies should be facilitated in the near future by the availability of humanized monoclonal antibodies designed to block GDF15 action.

## Notes

***Financial Statement:*** AM holds a PhD studentship at the University of Cambridge, which is supported jointly by the University of Cambridge Experimental Medicine Training Initiative (EMI) program in partnership with AstraZeneca. GJ and DC are supported by the Swedish Research Council (Project 2015–02561) and Swedish Federal Government under the LUA/ALF agreement (Project ALFGBG-719531). RHS is supported by the Medical Research Council (MR/K010271/1) and Chief Scientist Office (SCAF/17/02). BRW acknowledges support from Wellcome Trust (107049/Z/15/Z) and British Heart Foundation (RG/11/4/28734). SOR acknowledges support from the Wellcome Trust (WT 214274/Z/18/Z), the Medical Research Council (MRC Metabolic Disease Unit–MRC_MC_UU_12012.1), and the National Institute for Health Research (NIHR) Cambridge Biomedical Research Centre and NIHR Rare Disease Translational Research Collaboration.

## Additional Information

***Disclosure Summary:*** The authors declare no conflict of interest that is relevant to the subject matter or materials included in this work.
